# Special Issue “Chemical Speciation of Organic and Inorganic Components of Environmental and Biological Interest in Natural Fluids: Behaviour, Interaction and Sequestration”

**DOI:** 10.3390/molecules25040826

**Published:** 2020-02-13

**Authors:** Francesco Crea, Alberto Pettignano

**Affiliations:** 1 Dipartimento di Scienze Chimiche, Università di Messina, Viale Ferdinando Stagno d’Alcontres, 31, I-98166 Messina (Vill. S. Agata), Italy; 2 Dipartimento di Fisica e Chimica, Università di Palermo, Viale delle Scienze, I-90128 Palermo, Italy

Several different definitions were in the past proposed to describe the term chemical speciation, and some of them were accepted from the scientific community. Some examples of such definitions are as follows: “Chemical speciation can be defined as the process of identification and quantification of the different forms or phases in which an element is present in a material” or as “the description of quantities, types of species, forms or phases present in a material” [1,2].

Many authors used the term chemical speciation to explain different reaction types: (i) the chemical reactions that transform a set of metal compounds in a sample into another set of components, (ii) the assembly of compounds containing a given component that are present in a sample, and (iii) the process of identification and quantification of the metallic species present in a sample. 

The International Union of Pure and Applied Chemistry (IUPAC) sums up that “speciation” denotes to “the distribution of an element amongst defined chemical species in a system”, while the process leading to the quantitative estimation of the content of different species is called speciation analysis [3], as also reported in Ref. [4].

Over the last 2–3 decades, an increase in interest from chemists, biochemists, and biologists in techniques for chemical speciation studies has been observed, since it is now established that both bioavailability and toxicity are critically dependent on the chemical form of the given element in a given environment. 

The chemical speciation now involves various sectors of the sciences, from chemistry, to biology, to biochemistry, to environmental sciences, since, as it is well known, the total concentration of an inorganic or organic component (metal or ligand) in a multicomponent natural system (fresh water, sea water, biological fluids, soil, etc.) provides insufficient information to deeply understand its behavior in those contests. 

Biochemical and toxicological investigation has shown that, for living organisms, the chemical form of a specific element, or the oxidation state in which that element is introduced into the environment, is crucial, as well as the quantities [5]. Therefore, to get information on the activity of specific elements in the environment, more particularly for those in contact with living organisms, it is necessary to determine not only the total content of the element but also to gain an indication of its individual chemical and physical form.

As an example, in the case of metal toxicity, it is generally accepted that the free (hydrated) metal ion is the form most toxic to aquatic life. Strongly complexed metal, or metal associated with colloidal particles, is much less toxic [6]. 

Chemical speciation has been presenting great relevance, leading to the development of various methods of analysis used in the areas of health, food quality control, and the environment. 

Metals and metalloids are present in all compartments of our environment and the environmental pathways of these elements are of high importance in relation to their toxicity towards flora and fauna. Their concentration levels, mobility, and transformation and accumulation processes in the ecosystem depend on parameters such as pH, redox conditions, oxidation states, temperature, the presence of organic matter, and microbiological activity. All these factors strongly influence the biogeochemical cycles of elements in our environment.

For this reason, as guest editors, we thought about proposing the Special Issue: “Chemical Speciation of Organic and Inorganic Components of Environmental and Biological Interest in Natural Fluids: Behaviour, Interaction, and Sequestration” in the Molecules journal. The primary goal was to involve scientists from different sectors who could propose scientific contributions that, even with different approaches, involve the sector of chemical speciation and speciation analysis.

The Special Issue had satisfactory feedback from researchers, with contributions having cross-field character and being of interest for different industrial, pharmaceutical, chemical, and biological fields [7,8,9,10,11,12,13,14,15,16,17].

The Special Issue is accessible through the following link: https://www.mdpi.com/journal/molecules/special_issues/chemical_speciation_natural_fluids

As guest editors for this Special Issue, we would like to thank all the authors and co-authors for their contributions and all the reviewers for their efforts in carefully evaluating the manuscripts. Moreover, we would like to appreciate the editorial office of the Molecules journal for their kind assistance in preparing this Special Issue and, in particular, Ms. Katie Zhang, managing editor of the “Analytical Chemistry” section, for her precious help during the various stages of the organization and programming of the Special Issue.
